# Supplementary material for: Pyruvate Dehydrogenase Kinase 1 inhibition mediated oxidative phosphorylation enhancement in cartilage promotes osteoarthritis progression
Source: BMC Musculoskelet Disord. 2023 Jul 20;24:597. doi: 10.1186/s12891-023-06585-6 (PMC10357736; doi:10.1186/s12891-023-06585-6)
Supplement: Supplementary file 1 — Additional file 1. [file 12891_2023_6585_MOESM1_ESM.zip › KEGG.Gsea.1653622432464/KEGG_RIBOSOME.html]

Details for gene set KEGG\_RIBOSOME[GSEA]

|  || Dataset | 55457\_collapsed\_to\_symbols.cls.cls #OA\_versus\_normal.cls.cls #OA\_versus\_normal\_repos |
| Phenotype | cls.cls#OA\_versus\_normal\_repos |
| Upregulated in class | normal |
| GeneSet | KEGG\_RIBOSOME |
| Enrichment Score (ES) | -0.66434026 |
| Normalized Enrichment Score (NES) | -2.7843614 |
| Nominal p-value | 0.0 |
| FDR q-value | 0.0 |
| FWER p-Value | 0.0 |
Table: GSEA Results Summary

  

Fig 1: Enrichment plot: KEGG\_RIBOSOME      
 Profile of the Running ES Score & Positions of GeneSet Members on the Rank Ordered List

  

| SYMBOL | TITLE | RANK IN GENE LIST | RANK METRIC SCORE | RUNNING ES | CORE ENRICHMENT || 1 | MRPL13 | mitochondrial ribosomal protein L13 [Source:HGNC Symbol;Acc:HGNC:14278] | 1655 | 0.406 | -0.1177 | No |
| 2 | RPL36AL | ribosomal protein L36a like [Source:HGNC Symbol;Acc:HGNC:10346] | 2255 | 0.332 | -0.1552 | No |
| 3 | RPL36A | ribosomal protein L36a [Source:HGNC Symbol;Acc:HGNC:10359] | 3387 | 0.213 | -0.2375 | No |
| 4 | RPL18 | ribosomal protein L18 [Source:HGNC Symbol;Acc:HGNC:10310] | 3749 | 0.182 | -0.2605 | No |
| 5 | RPS27L | ribosomal protein S27 like [Source:HGNC Symbol;Acc:HGNC:18476] | 3882 | 0.166 | -0.2662 | No |
| 6 | RPL18A | ribosomal protein L18a [Source:HGNC Symbol;Acc:HGNC:10311] | 4049 | 0.151 | -0.2749 | No |
| 7 | FAU | FAU ubiquitin like and ribosomal protein S30 fusion [Source:HGNC Symbol;Acc:HGNC:3597] | 4418 | 0.119 | -0.3002 | No |
| 8 | RPL26L1 | ribosomal protein L26 like 1 [Source:HGNC Symbol;Acc:HGNC:17050] | 4584 | 0.106 | -0.3101 | No |
| 9 | RPL10A | ribosomal protein L10a [Source:HGNC Symbol;Acc:HGNC:10299] | 4974 | 0.074 | -0.3384 | No |
| 10 | UBA52 | ubiquitin A-52 residue ribosomal protein fusion product 1 [Source:HGNC Symbol;Acc:HGNC:12458] | 4978 | 0.074 | -0.3366 | No |
| 11 | RPS26 | ribosomal protein S26 [Source:HGNC Symbol;Acc:HGNC:10414] | 5368 | 0.043 | -0.3657 | No |
| 12 | RPS19 | ribosomal protein S19 [Source:HGNC Symbol;Acc:HGNC:10402] | 5952 | -0.002 | -0.4112 | No |
| 13 | RSL24D1 | ribosomal L24 domain containing 1 [Source:HGNC Symbol;Acc:HGNC:18479] | 5992 | -0.004 | -0.4141 | No |
| 14 | RPL15 | ribosomal protein L15 [Source:HGNC Symbol;Acc:HGNC:10306] | 6185 | -0.019 | -0.4285 | No |
| 15 | RPL35A | ribosomal protein L35a [Source:HGNC Symbol;Acc:HGNC:10345] | 6383 | -0.034 | -0.4430 | No |
| 16 | RPL10L | ribosomal protein L10 like [Source:HGNC Symbol;Acc:HGNC:17976] | 6502 | -0.042 | -0.4510 | No |
| 17 | RPL8 | ribosomal protein L8 [Source:HGNC Symbol;Acc:HGNC:10368] | 6606 | -0.050 | -0.4576 | No |
| 18 | RPL12 | ribosomal protein L12 [Source:HGNC Symbol;Acc:HGNC:10302] | 6845 | -0.067 | -0.4743 | No |
| 19 | RPL36 | ribosomal protein L36 [Source:HGNC Symbol;Acc:HGNC:13631] | 6860 | -0.068 | -0.4735 | No |
| 20 | RPS27A | ribosomal protein S27a [Source:HGNC Symbol;Acc:HGNC:10417] | 6973 | -0.078 | -0.4800 | No |
| 21 | RPL35 | ribosomal protein L35 [Source:HGNC Symbol;Acc:HGNC:10344] | 7563 | -0.124 | -0.5225 | No |
| 22 | RPL3L | ribosomal protein L3 like [Source:HGNC Symbol;Acc:HGNC:10351] | 8097 | -0.163 | -0.5595 | No |
| 23 | RPS21 | ribosomal protein S21 [Source:HGNC Symbol;Acc:HGNC:10409] | 8394 | -0.185 | -0.5774 | No |
| 24 | RPS9 | ribosomal protein S9 [Source:HGNC Symbol;Acc:HGNC:10442] | 8508 | -0.194 | -0.5808 | No |
| 25 | RPL29 | ribosomal protein L29 [Source:HGNC Symbol;Acc:HGNC:10331] | 8661 | -0.205 | -0.5869 | No |
| 26 | RPSA | ribosomal protein SA [Source:HGNC Symbol;Acc:HGNC:6502] | 8866 | -0.222 | -0.5966 | No |
| 27 | RPL28 | ribosomal protein L28 [Source:HGNC Symbol;Acc:HGNC:10330] | 8985 | -0.230 | -0.5993 | No |
| 28 | RPLP2 | ribosomal protein lateral stalk subunit P2 [Source:HGNC Symbol;Acc:HGNC:10377] | 9185 | -0.246 | -0.6080 | No |
| 29 | RPL37 | ribosomal protein L37 [Source:HGNC Symbol;Acc:HGNC:10347] | 9265 | -0.253 | -0.6070 | No |
| 30 | RPS25 | ribosomal protein S25 [Source:HGNC Symbol;Acc:HGNC:10413] | 9282 | -0.254 | -0.6012 | No |
| 31 | RPL4 | ribosomal protein L4 [Source:HGNC Symbol;Acc:HGNC:10353] | 9770 | -0.298 | -0.6308 | No |
| 32 | RPL5 | ribosomal protein L5 [Source:HGNC Symbol;Acc:HGNC:10360] | 10201 | -0.341 | -0.6548 | Yes |
| 33 | RPL24 | ribosomal protein L24 [Source:HGNC Symbol;Acc:HGNC:10325] | 10263 | -0.347 | -0.6498 | Yes |
| 34 | RPL22 | ribosomal protein L22 [Source:HGNC Symbol;Acc:HGNC:10315] | 10270 | -0.348 | -0.6405 | Yes |
| 35 | RPS8 | ribosomal protein S8 [Source:HGNC Symbol;Acc:HGNC:10441] | 10525 | -0.376 | -0.6498 | Yes |
| 36 | RPL31 | ribosomal protein L31 [Source:HGNC Symbol;Acc:HGNC:10334] | 10533 | -0.377 | -0.6398 | Yes |
| 37 | RPS7 | ribosomal protein S7 [Source:HGNC Symbol;Acc:HGNC:10440] | 10608 | -0.384 | -0.6348 | Yes |
| 38 | RPL10 | ribosomal protein L10 [Source:HGNC Symbol;Acc:HGNC:10298] | 10684 | -0.391 | -0.6297 | Yes |
| 39 | RPS11 | ribosomal protein S11 [Source:HGNC Symbol;Acc:HGNC:10384] | 10734 | -0.397 | -0.6223 | Yes |
| 40 | RPL17 | ribosomal protein L17 [Source:HGNC Symbol;Acc:HGNC:10307] | 10806 | -0.406 | -0.6165 | Yes |
| 41 | RPL7A | ribosomal protein L7a [Source:HGNC Symbol;Acc:HGNC:10364] | 10811 | -0.406 | -0.6054 | Yes |
| 42 | RPS4X | ribosomal protein S4 X-linked [Source:HGNC Symbol;Acc:HGNC:10424] | 10812 | -0.406 | -0.5940 | Yes |
| 43 | RPS29 | ribosomal protein S29 [Source:HGNC Symbol;Acc:HGNC:10419] | 10913 | -0.419 | -0.5901 | Yes |
| 44 | RPS4Y1 | ribosomal protein S4 Y-linked 1 [Source:HGNC Symbol;Acc:HGNC:10425] | 10932 | -0.422 | -0.5796 | Yes |
| 45 | RPL13 | ribosomal protein L13 [Source:HGNC Symbol;Acc:HGNC:10303] | 11013 | -0.431 | -0.5738 | Yes |
| 46 | RPL38 | ribosomal protein L38 [Source:HGNC Symbol;Acc:HGNC:10349] | 11122 | -0.446 | -0.5697 | Yes |
| 47 | RPL6 | ribosomal protein L6 [Source:HGNC Symbol;Acc:HGNC:10362] | 11129 | -0.447 | -0.5576 | Yes |
| 48 | RPL14 | ribosomal protein L14 [Source:HGNC Symbol;Acc:HGNC:10305] | 11154 | -0.451 | -0.5469 | Yes |
| 49 | RPS5 | ribosomal protein S5 [Source:HGNC Symbol;Acc:HGNC:10426] | 11206 | -0.458 | -0.5380 | Yes |
| 50 | RPS3 | ribosomal protein S3 [Source:HGNC Symbol;Acc:HGNC:10420] | 11245 | -0.463 | -0.5280 | Yes |
| 51 | RPL11 | ribosomal protein L11 [Source:HGNC Symbol;Acc:HGNC:10301] | 11252 | -0.464 | -0.5154 | Yes |
| 52 | RPS10 | ribosomal protein S10 [Source:HGNC Symbol;Acc:HGNC:10383] | 11408 | -0.488 | -0.5138 | Yes |
| 53 | RPL32 | ribosomal protein L32 [Source:HGNC Symbol;Acc:HGNC:10336] | 11447 | -0.496 | -0.5029 | Yes |
| 54 | RPS20 | ribosomal protein S20 [Source:HGNC Symbol;Acc:HGNC:10405] | 11507 | -0.502 | -0.4934 | Yes |
| 55 | RPL21 | ribosomal protein L21 [Source:HGNC Symbol;Acc:HGNC:10313] | 11647 | -0.524 | -0.4896 | Yes |
| 56 | RPS6 | ribosomal protein S6 [Source:HGNC Symbol;Acc:HGNC:10429] | 11662 | -0.526 | -0.4759 | Yes |
| 57 | RPS15 | ribosomal protein S15 [Source:HGNC Symbol;Acc:HGNC:10388] | 11671 | -0.527 | -0.4618 | Yes |
| 58 | RPS16 | ribosomal protein S16 [Source:HGNC Symbol;Acc:HGNC:10396] | 11755 | -0.541 | -0.4531 | Yes |
| 59 | RPL27 | ribosomal protein L27 [Source:HGNC Symbol;Acc:HGNC:10328] | 11765 | -0.544 | -0.4385 | Yes |
| 60 | RPL19 | ribosomal protein L19 [Source:HGNC Symbol;Acc:HGNC:10312] | 11967 | -0.587 | -0.4377 | Yes |
| 61 | RPS27 | ribosomal protein S27 [Source:HGNC Symbol;Acc:HGNC:10416] | 12071 | -0.612 | -0.4286 | Yes |
| 62 | RPS17 | ribosomal protein S17 [Source:HGNC Symbol;Acc:HGNC:10397] | 12130 | -0.626 | -0.4156 | Yes |
| 63 | RPL30 | ribosomal protein L30 [Source:HGNC Symbol;Acc:HGNC:10333] | 12140 | -0.629 | -0.3986 | Yes |
| 64 | RPS12 | ribosomal protein S12 [Source:HGNC Symbol;Acc:HGNC:10385] | 12156 | -0.632 | -0.3821 | Yes |
| 65 | RPL13A | ribosomal protein L13a [Source:HGNC Symbol;Acc:HGNC:10304] | 12200 | -0.645 | -0.3673 | Yes |
| 66 | RPS24 | ribosomal protein S24 [Source:HGNC Symbol;Acc:HGNC:10411] | 12223 | -0.652 | -0.3508 | Yes |
| 67 | RPLP0 | ribosomal protein lateral stalk subunit P0 [Source:HGNC Symbol;Acc:HGNC:10371] | 12285 | -0.672 | -0.3367 | Yes |
| 68 | RPS15A | ribosomal protein S15a [Source:HGNC Symbol;Acc:HGNC:10389] | 12291 | -0.674 | -0.3182 | Yes |
| 69 | RPS13 | ribosomal protein S13 [Source:HGNC Symbol;Acc:HGNC:10386] | 12334 | -0.687 | -0.3022 | Yes |
| 70 | RPL3 | ribosomal protein L3 [Source:HGNC Symbol;Acc:HGNC:10332] | 12350 | -0.691 | -0.2840 | Yes |
| 71 | RPLP1 | ribosomal protein lateral stalk subunit P1 [Source:HGNC Symbol;Acc:HGNC:10372] | 12467 | -0.734 | -0.2725 | Yes |
| 72 | RPL7 | ribosomal protein L7 [Source:HGNC Symbol;Acc:HGNC:10363] | 12519 | -0.757 | -0.2552 | Yes |
| 73 | RPS28 | ribosomal protein S28 [Source:HGNC Symbol;Acc:HGNC:10418] | 12526 | -0.761 | -0.2344 | Yes |
| 74 | RPS2 | ribosomal protein S2 [Source:HGNC Symbol;Acc:HGNC:10404] | 12541 | -0.769 | -0.2139 | Yes |
| 75 | RPL23 | ribosomal protein L23 [Source:HGNC Symbol;Acc:HGNC:10316] | 12558 | -0.777 | -0.1934 | Yes |
| 76 | RPL27A | ribosomal protein L27a [Source:HGNC Symbol;Acc:HGNC:10329] | 12592 | -0.792 | -0.1737 | Yes |
| 77 | RPL34 | ribosomal protein L34 [Source:HGNC Symbol;Acc:HGNC:10340] | 12602 | -0.799 | -0.1520 | Yes |
| 78 | RPS18 | ribosomal protein S18 [Source:HGNC Symbol;Acc:HGNC:10401] | 12611 | -0.804 | -0.1301 | Yes |
| 79 | RPS23 | ribosomal protein S23 [Source:HGNC Symbol;Acc:HGNC:10410] | 12661 | -0.834 | -0.1105 | Yes |
| 80 | RPL41 | ribosomal protein L41 [Source:HGNC Symbol;Acc:HGNC:10354] | 12674 | -0.847 | -0.0877 | Yes |
| 81 | RPL37A | ribosomal protein L37a [Source:HGNC Symbol;Acc:HGNC:10348] | 12754 | -0.918 | -0.0681 | Yes |
| 82 | RPS3A | ribosomal protein S3A [Source:HGNC Symbol;Acc:HGNC:10421] | 12758 | -0.921 | -0.0426 | Yes |
| 83 | RPL23A | ribosomal protein L23a [Source:HGNC Symbol;Acc:HGNC:10317] | 12785 | -0.944 | -0.0181 | Yes |
| 84 | RPL39 | ribosomal protein L39 [Source:HGNC Symbol;Acc:HGNC:10350] | 12797 | -0.963 | 0.0080 | Yes |
Table: GSEA details [plain text format]

  

Fig 2: KEGG\_RIBOSOME      
 Blue-Pink O' Gram in the Space of the Analyzed GeneSet

  

Fig 3: KEGG\_RIBOSOME: Random ES distribution      
 Gene set null distribution of ES for **KEGG\_RIBOSOME**
